# Effect of concrete slats, three mat types and out-wintering pads on performance and welfare of finishing beef steers

**DOI:** 10.1186/s13028-017-0302-3

**Published:** 2017-05-30

**Authors:** Bernadette Earley, John D. McNamara, Stephen J. Jerrams, Edward G. O’Riordan

**Affiliations:** 1 Animal and Bioscience Research Department, Animal & Grassland Research and Innovation Centre, Teagasc, Grange, Dunsany, Co. Meath, C15 PW93 Ireland; 2 Centre for Elastomer Research (CER), School of Manufacturing and Design Engineering, Dublin Institute of Technology (DIT), Dublin, Ireland; 3 Livestock Systems Research, Department Animal & Grassland Research and Innovation Centre, Teagasc, Grange, Dunsany, Co. Meath, C15 PW93 Ireland

**Keywords:** Housing, Animal welfare, Concrete slat, Rubber mat

## Abstract

**Background:**

The objective was to investigate the effect of placing mats on concrete slatted floors on performance, behaviour, hoof condition, dirt scores, physiological and immunological variables of beef steers, and to compare responses with animals on out-wintering pads. Continental crossbred beef steers [n = 360; mean (±SD) initial live weight 539 kg (42.2)] were blocked by breed and live weight and randomly assigned to one of five treatments; (1) Concrete slats alone, (2) Mat 1 (Natural Rubber structure) (Durapak Rubber Products), (3) Mat 2 (Natural rubber structure) (EasyFix), (4) Mat 3 (modified ethylene vinyl acetate (EVA) foam structure) and (5) Out-wintering pads (OWP’s).

**Results:**

Animals on the OWPs had a greater (P < 0.05) live weight gain (P < 0.05) compared with the slat and Mat 2 treatments: results for Mat 1 and Mat 3 were the same (P > 0.05) as the other treatments. Animals on the OWPs had reduced lying percentage time compared with all the other treatments. Dry matter (DM) intake was greater for animals on the OWPs compared with all the other treatments. Carcass weight, kill out proportion, carcass fat score, carcass composition score, FCR and physiological responses were similar (P > 0.05) among treatments. No incidence of laminitis was observed among treatments. The number of hoof lesions was greater on all mat types (P < 0.05) compared with concrete slats and OWP treatments. Dirt scores were greater (P < 0.05) for animals on OWPs when measured on days 42, 84, 105, 126 and 150 compared with animals on slats.

**Conclusions:**

Under the conditions adopted for the present study, there was no evidence to suggest that animals housed on bare concrete slats were disadvantaged in respect of animal welfare compared with animals housed on other floor types. It is concluded that the welfare of steers was not adversely affected by slats compared with different mat types or OWPs.

## Background

There is a shortage of published research on the effect of placing mats on concrete slats in facilities for winter finishing beef cattle. Most previous housing studies reported significant behavioural alterations concerning the welfare of cattle accommodated on fully slatted concrete floors [1–9]. Elmore et al. [1] reported that the addition of slatted rubber mats to concrete pens improved locomotion, leg and joint health, and alterations in behaviour that are indicative of increased traction and reduced discomfort in finishing beef steers. Other studies have measured the growth of finishing cattle in response to a range of space allowances [10–13]. Biological performance is an indicator of animal well-being and previous studies have demonstrated that stress due to reduced space allowance can negatively affect animal performance [13–16]. Schlichting et al. and Schlichting and Smidt [15, 16] reported that the abrasive effect of the concrete slatted floor led to reduced slipping of hooves on contact. A study by Murphy et al. [17] measured the in vitro abrasion on a wear-testing instrument of hoof horn samples of cattle in relation to breed and housing. The hoof horn of Friesian cattle was softer if they had been housed on concrete slats rather than on straw, but there was no such effect in Hereford cattle. Interest in out-wintering pads (OWPs) as a low cost alternative to indoor housing is another system that has been studied [18]. The OWP’s consist of a woodchip lying area constructed over a drainage system, which may or may not be sheltered. Animals have access to feed from a self-feeder at the feed-face of the OWP. The OWP system has been shown, under experimental conditions, to meet environmental guidelines as well as being capable of supporting satisfactory animal performance, health and welfare [18].

The hypothesis of the study was that the provision of a Mat flooring or an out-wintering pad (OWP) would offer protection to the animals’ feet, particularly over a 150-day winter period, which would in turn improve hoof health. Furthermore, it was hypothesised that finishing cattle on the mats or OWPs would have enhanced performance compared with concrete slats owing to more comfortable underfoot conditions that would in turn lead to improvements in animal welfare. The objective was to investigate the effect of placing mats on concrete slatted floors on performance, hoof condition and body cleanliness of finishing beef steers, and to compare responses with animals on out-wintering pads. Accordingly, this study examined the behavioural, physiological and immunological responses of the steers by assessing the consequences of the floor type on the welfare of finishing beef cattle that were offered a total mixed ration (TMR) diet.

## Methods

### Environmental conditions

This study was conducted at Teagasc Grange, located in Co. Meath, Ireland (latitude 53.52187, longitude-6.65247). The mean daily air temperature in the housing facility (from November to May) and the ambient environment was continuously recorded using Testo 175 data loggers (Radionics, Dublin, Ireland).

### Animals, experimental design and diet

Continental crossbred beef steers [n = 360; mean (±SD) initial live-weight 539 kg (42.2)] were blocked by breed and live-weight and randomly assigned to one of five treatments; (1) Concrete slats alone, (2) Mat 1 (Natural Rubber structure) (Durapak Rubber Products, Cork, Ireland), (3) Mat 2 (Natural rubber structure) (EasyFix, Galway, Ireland), (4) Mat 3 [modified ethylene vinyl acetate (EVA) foam structure (Mayo, Ireland)] and (5) Out-wintering pads (OWPs) (constructed in 2006). Animals assigned to treatments 1–4 were accommodated in a roofed building with concrete slatted floors. The concrete slats (5 gang/pen) in the pens were supplied by Banagher Concrete Ltd., Offaly, Ireland. Each gang slat had the following dimensions; void length 945 mm × 3 mm; void space 35 mm; slat length 3.5 m; slat rib width 170 mm; slat width 1.18 m. The mats were supplied and fitted to the concrete slats in the cattle pens by the respective suppliers. There were eight pens per treatment, with nine steers per pen at a mean space allowance of 2.73 m^2^/head, whereas animals accommodated outdoors on the OWPs had a space allowance of 12 m^2^/head. The shed was dimly lit using infra-red lighting to allow visualisation during the behavioural studies.

### Animal diet

All animals were fed a total mixed ration (TMR) of silage and rolled barley on a 50:50 dry matter (DM) basis. Typically 2000 kg of silage and 500 kg rolled barley was mixed in a 12 cubic meter Abbey Vertical Mixer (VF 12) fitted with a Digistar weighing system. Feed was weighed into each group every day at 9:00 throughout the study and refusals were measured twice weekly. The steers had free access to water drinkers in their pens. Each pen was fitted with a single 2 l nose fill water bowl which was positioned 55 cm above the floor of the pen. The TMR samples offered were submitted to Grange Labs at Teagasc for chemical analysis [dry matter digestibility (DMD), crude protein (CP), ash, neutral detergent fibre (NDF)] and pH analyses [19]. Animals were vaccinated with a Covexin 8 (Merck, Animal Health, MSD Animal Health, Dublin, Ireland) and a Bovilis-Bovipast RSP (Merck, Animal Health, MSD Animal Health, Dublin, Ireland) injections 3 weeks prior to commencement of the study.

### Health

The general health status of the steers was recorded by a trained staff member. A complete record of any clinical symptoms by infection or injury and their veterinary treatment was maintained.

### Hoof condition

The general condition of the four hooves of each animal was recorded prior to (day 0) and at the end of the study (i.e., on retrieval of the hooves post-slaughter of the animals). A single observer, who was experienced in hoof examination assessed the four hooves of each animal on both occasions and preliminary scoring was carried out to verify repeatability of results. The hooves were trimmed on day 0 and again on the retrieval of the hooves post-slaughter, to allow visualization. Both claws per each of the four hooves were examined by the same trained observer for the presence of lesions using the method of Greenough and Vermunt [20]. The dorsal area was examined for equal and unequal size and the plantar area for heel erosion, under run sole, digital dermatitis, inter-digital dermatitis and white line damage. The total number of lesions was averaged over all four hooves and the number each animal had obtained during the study was determined. Animals were observed for any signs of lameness every 3 weeks to coincide with animal live weight recordings. All animals were assessed by the same trained person for abnormal gait and back posture while walking and standing using the method of Sprecher et al. [21] on a scale of 1 (normal) to 5 (abnormally lame).

#### Animal cleanliness scoring

All animals were dirt scored on day 0 before assignment to treatment and at 21 day intervals (day 0, 21, 42, 63, 84, 105, 126 and 150) throughout the study. Dirt scoring was carried out by the same person on each occasion and was carried out blind to treatment. The body dirt scoring system used by Earley et al. [22], which was adapted from Scott and Kelly [23] was applied. Using this system, the entire left side of each animal was diagrammatically divided into 16 body segments and each segment was assigned a score between 1 (very clean) and 5 (very dirty) levels of intensity. Each animal was then given an overall dirt score between 16 and 80, which was equal to the sum of the scores for each of the 16 body segments [22].

### Behaviour

An individual Eneo CCTV camera was placed over each pen and the behavioural data were recorded. Behavioural observations were conducted over six 3-week intervals, continuously for 72 h duration. Animals were identified by their natural body markings. During the period of darkness, the shed housing the animals was dimly lit using infra-red lights to allow observation of the animals on the CCTV cameras. The cameras were connected to a video tape recorder (Panasonic AG6040) via a multi-vision system (Panasonic WJ-FS109, monochrome duplex multiplexer, Lynx, Dunsany, Co. Meath, Ireland) which allowed pictures from all cameras to be viewed on one screen at a time. The pictures from all the cameras were marked with individual pen number, time and date settings. The behavioural analysis was performed by a trained staff member. Steers were observed by instantaneous scan sampling of the CCTV recordings. The interval between scans was 10 min. This interval was chosen based on previous recording of animal behaviours [22, 24]. Each steer was observed for behavioural activity and body contact. Counts of lying, standing, eating and drinking, mounting, head-butting, licking and grooming were recorded. In the behavioural activity category, steers were observed for the following: lying down: head supported by the neck, head not supported by the neck (chin on the floor, on the body or on another steer); standing: with or without moving; eating (head in the trough); drinking; head to head contact except while eating; head contact with the body of another steer; and mounting. In the contact category the criteria were: no body contact with other steers and contact with one, two or three steers; grooming refers to self-grooming and allogrooming constitutes licking another animal.

### Average daily live weight and carcass characteristics

Live weights were measured on day-1 and again on day 0 before assignment to treatment, at 21-day intervals (day 0, 21, 42, 63, 84, 105, 126 and 150) throughout the study and average daily gain (ADG) determined. Animals were slaughtered from day 150 of the experiment onwards. Cold carcass weights were recorded, with carcass conformation and fat scores graded using a video imaging analysis carcass classification system (VBS 2000, E + P, Oranienberg, Germany) based on the EU Beef Carcass Classification Scheme according to the EU beef carcass classification scheme (EC, 2006) [25]. Feed conversion ratio (FCR) was expressed as kilograms of DMI/pen/day divided by kilograms of live weight gain/pen/day.

### Physiological, haematological and immunological variables

Animals were blood sampled via jugular venipuncture on days 0 and 150. For the blood sampling procedure, the animals were moved to a holding pen with a squeeze chute facility and were blood sampled with minimal restraint. Blood sampling was carried out by the same experienced operator on each occasion and the time taken to collect the blood samples was less than 60 s/animal.

#### Haematological measurements

Blood samples were collected into (1 × 6 ml) K_3_ ethylenediaminetetraacetic acid (K_3_EDTA) tube (Vacuette, Cruinn Diagnostics, Ireland) for haematological cell distributions and haemoglobin content determined using an automated analyser (Celltac MEK-6108 K; Nihon-Kohdon, Tokyo, Japan) and reagents supplied by Celltac (Alpha Technologies, Dublin, Ireland).

#### Blood metabolites, acute phase proteins and immune measurements

Heparinized blood samples were collected by jugular venipuncture and the plasma was separated by centrifugation at 1600×*g* at 8 °C for 15 min (except for interferon (IFN)-γ at 300×*g*; at 8 °C for 15 min) and subsequently stored at −20 °C until assayed for subsequent analysis of metabolic variables associated with mobilization of body energy reserves, dehydration and muscle injury [albumin, total protein, creatine kinase (CK), glucose, non-esterified fatty acid (NEFA), βeta-hydroxy butyrate (βHB)], inflammatory biomarkers (the acute phase proteins (fibrinogen, haptoglobin) and a measure of cell mediated immunity interferon-(IFN)γ production [26]).

#### Albumin

The concentration of albumin was determined on an automatic clinical analyser (Olympus AU400 Clinical Analyser, Tokyo, Japan) using the reagents supplied by Olympus (catalogue number OSR6102). The composition of the reagents (at final concentration) required for this test were: succinate buffer (pH 4.2) (100 mmol/l) and bromocresol green (0.2 mmol/l) and following gentle inversion the reagents were ready to use directly from the kit.

#### Total protein

The concentration of total protein in plasma was determined on an automatic clinical analyser (Olympus AU400 Clinical Analyser, Tokyo, Japan) using the reagents supplied by Olympus (catalogue number OSR6132) (Olympus UK Ltd., Voice House, Watford, Hertfordshire, WD24 4JL, UK). The composition of the reagents (at final concentration) required for this test were; sodium hydroxide (200 mmol/l), potassium sodium tartrate (32 mmol/l), copper sulphate (18.8 mmol/l) and potassium iodide (30 mmol/l) and following gentle inversion the reagents were ready to use directly from the kit.

#### Creatine kinase (CK)

The activity of CK was determined on an automatic clinical analyser (Olympus AU400 Clinical Analyser, Tokyo, Japan) using the reagents supplied by Olympus (catalogue number OSR6179). The composition of the reagents (at final concentrations of reactive ingredients) required for this test were: imidazole (pH 6.5) (100 mmol/l), NADP (2 mmol/l), ADP (2 mmol/l), AMP (5 mmol/l), EDTA (2 mmol/l), glucose (20 mmol/l), creatine phosphate (30 mmol/l), n-acetlycysteine (0.2 mmol/l), activator (26 mmol/l), Mg^2+^ (10 mmol/l), diadenosine pentaphosphate (0.01 mmol/l), hexokinase (>4 kU/l) and glucose-6-phosphate dehydrogenase (>2.8 kU/l). Prior to the placement of the reagents on board the instrument, the entire kit contents of R1-2 (4 ml) were transferred to the entire kit contents of R1-1 (22 ml) and mixed by gentle inversion. The second reagent bottle (R2) (6 ml) was ready to use from the kit and was placed directly on the instrument.

#### Glucose

The concentration of glucose was determined on an automatic clinical analyser (Olympus AU400 Clinical Analyser, Tokyo, Japan) using the reagents supplied by Olympus (catalogue number OSR6121). The composition of the reagents (at final concentration of reactive ingredients) required for this test were: piperazine-*N*,*N*’-bis (ethanesulfonic acid (PIPES) buffer (pH 7.6) (24 mmol/l)), ATP (≥2 mmol/l), NAD^+^ (≥1.32 mmol/l), Mg^2+^ (2.37 mmol/l), hexokinase (≥0.59 mmol/l), G6P-DH (≥0.59 mmol/l) and required no preparation prior to placement on the instrument.

#### Non-esterified fatty acids

The concentration of non-esterified fatty acids (NEFA) was determined on an automatic clinical analyser (Olympus AU400 Clinical Analyser, Tokyo, Japan) using the reagents supplied by Randox Laboratories (catalogue number FA115) (Randox Labs ltd., Ardmore, Diamond Rd., Crumlin BT29 4QY, Co. Antrim, Ireland). The kit comprised of five reagents. The buffer (R1a) and enzyme diluent (R2a) were ready to use from the kit. The enzyme/coenzymes (R1b) were reconstituted with 10 ml of buffer (R1a) and mixed by gently swirling. Maleimide (R2b) was reconstituted with enzyme diluent (R2a) and was inverted several times to ensure that maleimide was completely dissolved. This was then used immediately to reconstitute enzyme reagent (R2c) which was protected from light and placed on board the instrument for analysis. All reagents were kept at 4 °C prior to and after preparation.

#### β-Hydroxy butyrate (βHB)

The concentration of βHB was determined on an automatic clinical analyser (Olympus AU400 Clinical Analyser, Tokyo, Japan) using the reagents supplied by Randox Laboratories (catalogue number RB 1007). The kit comprised of five reagents: R1a [Buffer (Tris buffer (pH 8.5) 100 mmol/l), EDTA (2 mmol/l), Oxalic acid (20 mmol/l)] and R1b (Enzyme/Coenzyme NAD + 2.5 U/ml; 3-hydroxybutyrate dehydrogenase 0.12 U/ml)). The reagents were stored at 4 °C prior to and after preparation.

#### Acute phase protein response (fibrinogen and haptoglobin)

Blood collected into vacutainer tubes containing lithium heparin and sodium citrate was used to determine the plasma concentration of haptoglobin and fibrinogen, respectively. Plasma was harvested following centrifugation at 1600×*g* at 4 °C for 15 min and stored at −80 °C until assayed. Plasma concentration of haptoglobin was measured using an automatic analyser (spACE, Alfa Wassermann, Inc., West Caldwell, NJ, USA) and commercial assay kit (Tridelta Development Ltd., Wicklow, Ireland) according to the manufacturer’s procedure as described by Eckersall et al. [27]. Plasma concentration of fibrinogen was measured using an automatic analyser (spACE, Alfa Wassermann, Inc., West Caldwell, NJ, USA) using a method described by Becker et al. [28].

#### Immune variable; interferon-γ (IFN-γ)

Blood samples for IFN-γ determination were collected by jugular venipuncture into aseptic vacutainer tubes containing lithium heparin and the stimulated lymphocyte production of IFN-γ was determined following whole blood culture. Duplicate 1.48 ml aliquots of heparinized blood were cultured in sterile 24-well flat culture plates (Sarstedt Ltd., Drinagh, Wexford, Ireland) with 20 μl of PBS (GibcoBRL, Life Technologies Ltd., Paisley, Scotland) containing either 1 mg/ml of PHA (Sigma-Aldrich, Inc., St. Louis, MO, product No. L-9132) or 1 mg/ml of Concanavalin A (Con A; Sigma-Aldrich; product No. C 5275) or no additive for 24 h at 37 °C in an atmosphere of 5% CO_2_. Aseptic techniques were practised during this procedure under laminar flow conditions. The culture plates were then centrifuged at 1600×*g*, at 4 °C for 20 min; the supernatant was harvested and frozen at −20 °C until it was assayed for IFN-γ production, using an ELISA procedure [27] (Bovigam, CSL Biosciences, Victoria, Australia; catalogue No. 03000201). The in vitro phytohemagglutinin (PHA)- and Con A-stimulated IFN-γ production was calculated by subtracting the absorbance at 450 nm of wells that received PBS alone from the absorbance of wells that received either PHA or Con A [29].

#### Physical characteristics of the mats

The mats were tested to give an assessment of physical parameters: hardness, simulated hoof—mat contact properties and time dependent deformation characteristics at the German Institute for Rubber Technology, (Hannover [DIK]). The proprietary mats, hereafter referred to as Mats 1, 2, and 3, were compared. Testing conformed to current ISO international standards. Each mat had its own distinct pattern on the mat upper surface which would inevitably have had some minor influence on physical test results. Mats 1 and 2 were predominantly made of natural rubber (NR) and Mat 3 comprised a modified ethylene vinyl acetate (EVA) foam structure.

#### Shore A hardness

The Shore hardness test for rubber samples is basically a method of indirectly characterising the elastic modulus of a rubber by measuring the elastic indentation caused by a rigid indentor pressed into the surface under specified loading conditions. Theoretically, hardness values range from zero degrees when the modulus is zero, (very soft rubber) up to 100° where the modulus is infinitely high. The Shore A hardness of each sample was measured at room temperature and on a stand mounted digital Shore meter with a timer allowing the values of hardness to be determined consistently throughout testing. In each test a reading of hardness was taken after a 3 s constant force application of a standard indentor to a 6 mm thick specimen prepared in each instance from an actual mat. The values shown in Table 1 were an average of five tests, taken on different parts of the specimen prepared from the mat, but at least 12 mm from the edge of the specimen in all tests. This procedure complies with test standard ISO 7619:1997 (Rubber—determination of indentation hardness by means of pocket hardness meters).

**Table 1:** Results from the various tests that were used to characterise the physical properties of the mats

	Mat 1	Mat 2	Mat 3
Mean Shore A hardness (±SE)	64 (0.16)	70 (0.22)	61 (0.16)
Mean wet friction (μ)*	0.59	0.63	0.38
(Maximum)	(0.67)	(0.70)	(0.40)
(Minimum)	(0.53)	(0.57)	(0.37)
(±SE)	(0.04)	(0.04)	(0.01)
Mean dry friction (μ)*	0.71	0.74	0.40
(Maximum)	(0.74)	(0.76)	(0.43)
(Minimum)	(0.67)	(0.72)	(0.38)
(±SE)	(0.02)	(0.01)	(0.02)
Compression (mm)			
As result of applied stress equivalent to application of one hoof	0.839	1.090	0.933
Creep (mm)			
As result of an applied stress equivalent to the application of one hoof for 300 s	0.095	0.138	0.292

**Fig. 1:**
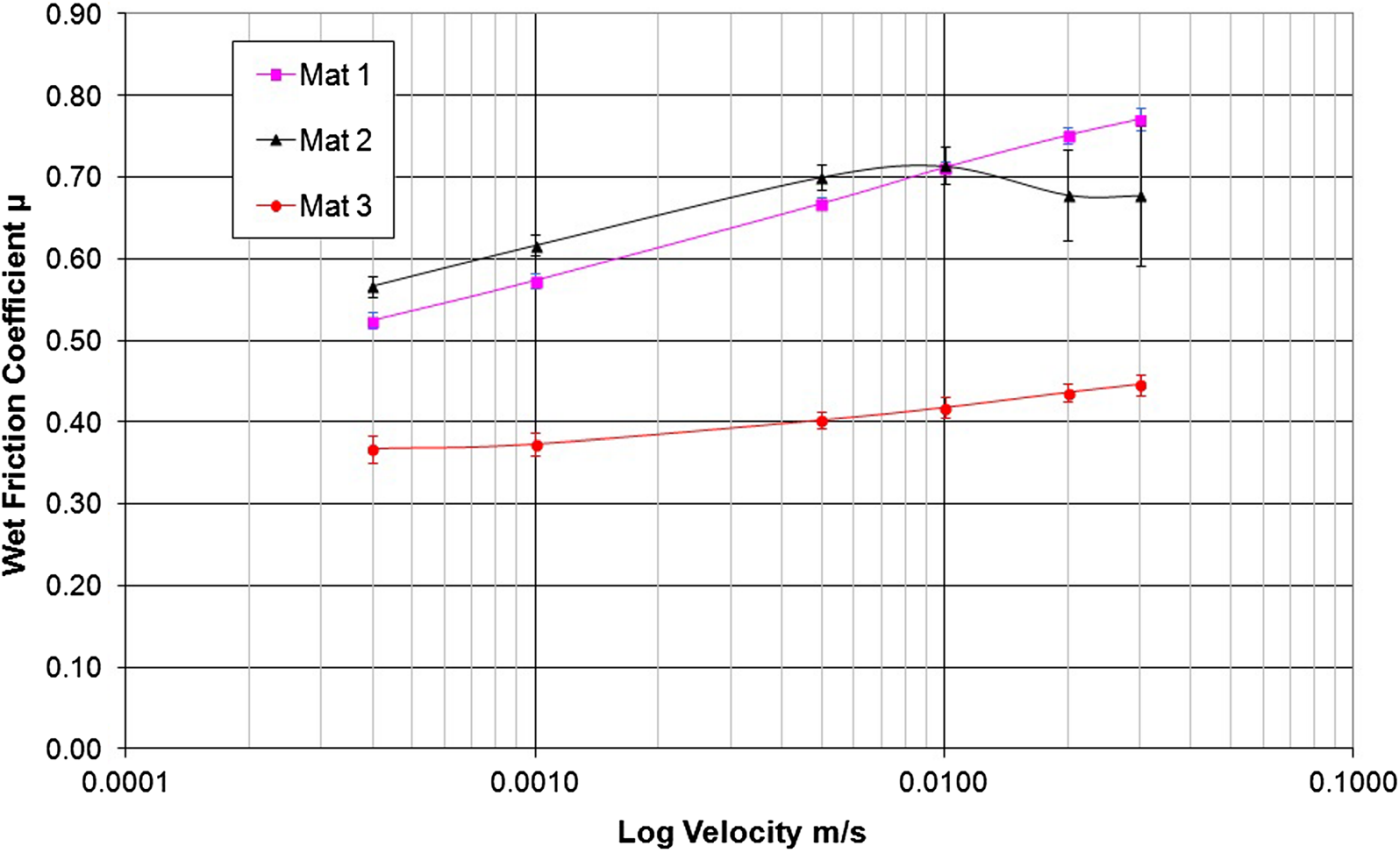
Wet friction values measured for a range of sliding velocities

**Fig. 2:**
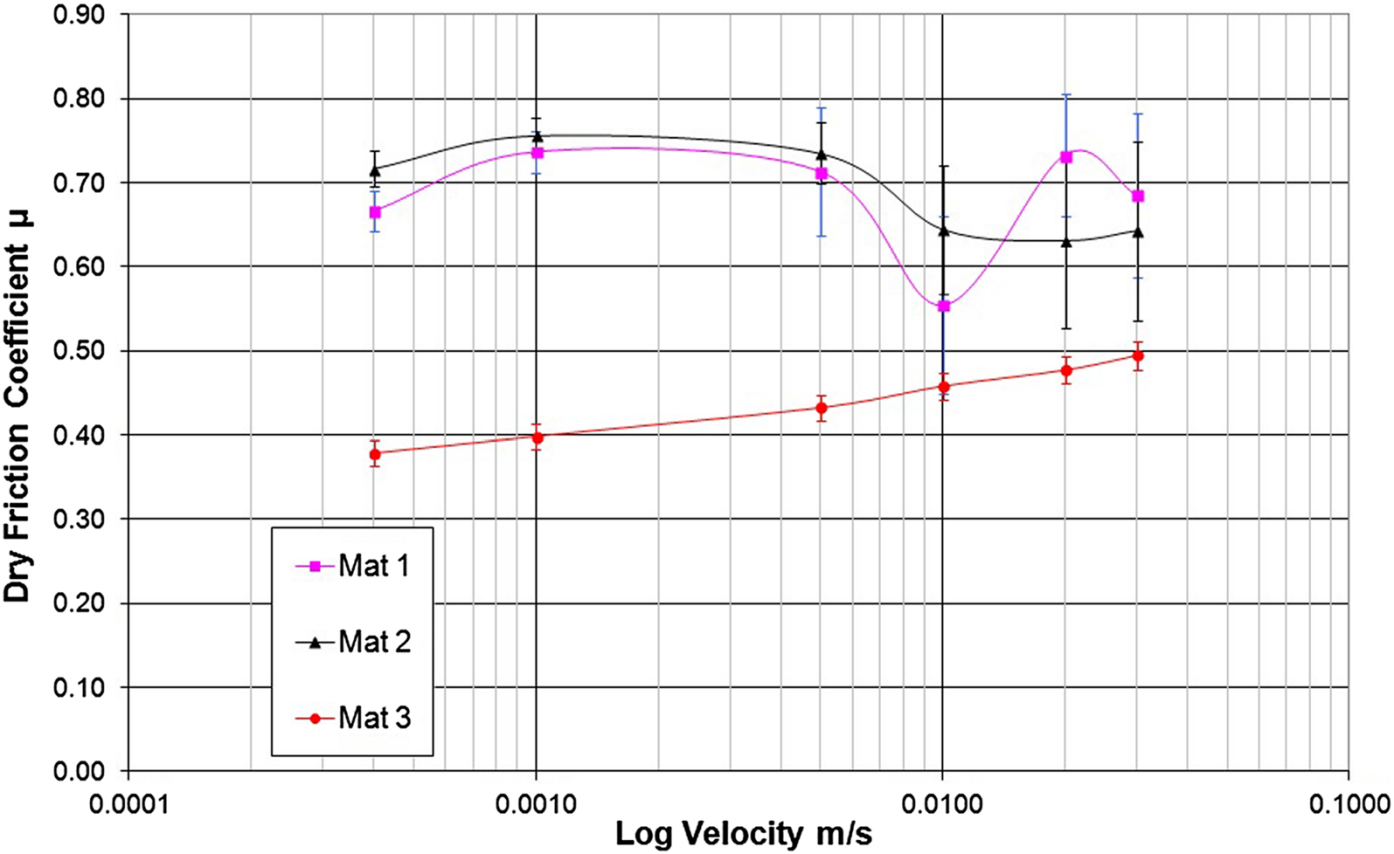
Dry friction values measured for a range of sliding velocities

* The mean values of μ displayed are calculated from the three lowest sliding velocities as illustrated in Figs. 1 and 2. This was conducted to eliminate the effect of the phenomenon known as “stick slip” which was a characteristic of the test apparatus rather than of the test materials

#### Friction measurements

The characterisation of friction coefficients was carried out using a Zwick universal testing machine (Type 1445.17, fitted with a 5 kN load cell) which was modified to encompass friction measurements. The tests were conducted over a range of sliding velocities from 0.1 to 30 mm/min and under an applied stress of 18.4 kPa. Under the range of sliding velocities any local interfacial thermal effects due to contact between the mat and the testing surface was negligible. Also, the amount of abrasion of the specimens was minimised by the selection of a low load.

The test specimens used were cut from actual mats and measured 40 × 40 mm^2^ with a thickness equal to the mat under test. The specimens were bonded to an aluminium alloy plate which was loaded with a 3 kg mass. The plate was then drawn by a stranded steel wire which traversed two pulleys, one of which was connected to the 5 kN load cell allowing the friction force to be measured directly. All tests were carried out at room temperature. For wet friction testing, a consistent level of wetness was ensured by the sample being immersed in a water/detergent (95:5) mixture for each test. Friction tests conformed to an adaptation of ISO 15113:1999, Rubber—determination of frictional properties.

#### Dry and wet friction

Samples of Mat 1, Mat 2 and Mat 3 were drawn over the test track surface. Initially the friction force peaked at a point where the adhesion force between the sample and surface began to break down and subsequently a slightly lower approximately constant frictional force was measured as the specimen travelled along the test track. This constant force is termed F_const_ and the friction coefficient was calculated from.

$$ \mu \, = {\text{ F}}_{\text{const}} /{\text{ F}}_{\text{N}} $$

where μ is the coefficient of friction and F_N_ is the normal force.

The test was repeated four times at each sliding velocity in planes normal to and in line with the mat surface patterns. An average friction coefficient was calculated for each material and sliding velocity (i.e. eight tests per quoted coefficient of friction).

#### Compression creep tests

The mats were subjected to a compression creep test on an MTS 831.50 elastomer testing system (2.5 kN load cell). This test was devised specifically to render useful data in respect of time dependent properties for each product. A force was applied through two flat plates, approximately equivalent to that which a finishing steer would typically exert on a mat due to the downward load transmitted through one hoof. This force was maintained for a period of 300 s to examine how the reaction force in the region of contact on the mat relaxed with time. The applied stress was calculated from an average animal mass (625 kg) divided by four and the surface area of a typical hoof (5300 mm^2^) as specified by Kirchner and Boxberger [30].

### Statistical analysis

All statistical analyses were performed using SAS software Version 9.3 (SAS Institute Inc. Cary, NC, USA). The pen was the experimental unit for all variables. Data were checked for normality and homogeneity of variance by histograms, q–q plots, and formal statistical tests as part of the UNIVARIATE procedure of SAS (version 9.3; SAS Institute, 2006). Data that were not normally distributed were transformed by raising the variable to the power of lambda. The appropriate lambda value was obtained by conducting a Box-Cox transformation analysis using the TRANSREG procedure of SAS. Data subjected to transformation were used to calculate P-values. Data from the three concrete slat treatments were analysed using a mixed model ANOVA with the MIXED procedure of SAS to examine the effect of treatment on intake, performance traits, hoof lesion scores, physiological and behavioural data. The PDIFF option was applied to evaluate the pair-wise comparisons between the OWP treatment and the concrete slat treatment. The statistical model included the fixed effect of treatment and batch. Data with multiple observations, such as dirt scores, haematological variables and metabolites, were analysed using a repeated measures ANOVA (MIXED procedure of SAS 9.3). Terms for treatment, day and their interaction were included in the model. If the interaction term was not significant (P > 0.05) it was subsequently excluded from the final model. The PDIFF option and the Tukey test were applied as appropriate to evaluate pair-wise comparisons between the group means on concrete slats and the associated P values were derived. Data were considered statistically significant when P < 0.05. Least square means (Lsmeans) are reported with standard error of the mean.

## Results

### Characteristics of mats

#### Shore A hardness

The mean (±SE) Shore A hardness for the three mat types were: Mat 1, 64 (0.16); Mat 2, 70 (0.22); Mat 3, 61 (0.16), respectively (Table 1).

#### Wet and dry friction results

A high friction coefficient for both wet and dry conditions is desirable between the hoof of the animal and the rubber mat. The higher the friction coefficient the less likely the animal is to slip when moving about in the pen, which will obviously provide a more comfortable environment for the animal. When the animal has firm and secure footing it will be more likely to move about its environment in a more natural manner to feed as desired without risk of slippage and sustaining injury.

The wet friction results are the most relevant for the normal environment encountered by the mats (Table 1). Figure 1 shows the results of the wet friction tests where, as stated previously, the test track was immersed in water with a 5% detergent mix. There is a clear difference in the friction coefficients for each material with some stick–slip behaviour having a pronounced influence, notably where friction coefficients for Mat 2 declined at greater sliding velocities.

Dry friction test results are shown in Table 1 and Fig. 2 and it was observed that Mats 1 and 2 experienced some stick–slip behaviour at greater velocities. Consistently in both the wet and dry friction tests, the ranking order was Mat 2, Mat 1 and Mat 3 (i.e. Mat 2 was shown to have the highest and Mat 3 the lowest coefficient of sliding friction in contact with a constant rigid surface). Thus Mat 2 exhibited superior friction properties to the other mats tested.

#### Compression creep tests

**Fig. 3:**
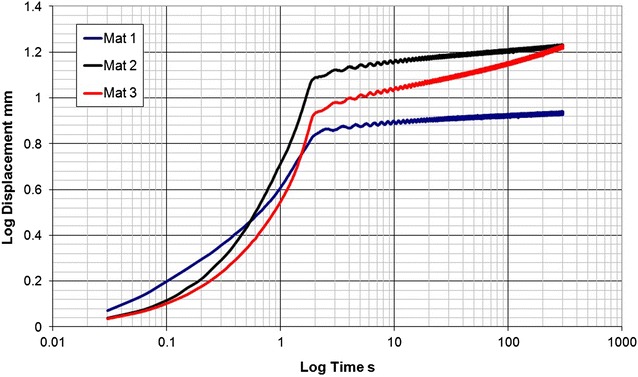
Graph showing results of compression tests where a stress equivalent to that exerted by a single hoof exerted upon the Mat

**Fig. 4:**
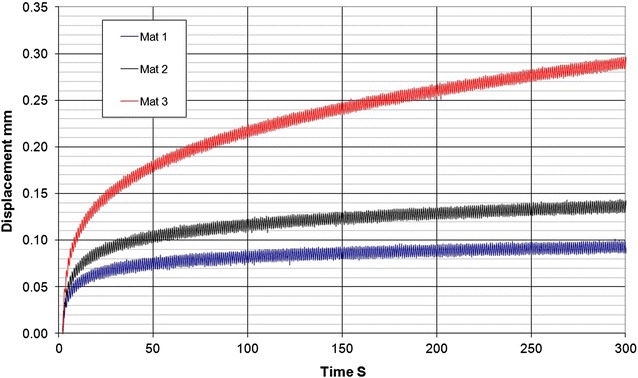
Graph showing the results of creep tests where a stress equivalent to that exerted by one hoof was maintained on the Mat for 300 s

The results from the compression creep tests are summarised in Fig. 3. Creep behaviour from a zero datum after the 2 s load application are presented in Fig. 4.

#### Environmental conditions

The mean (±SE) daily air temperature (^o^C) recorded in the shed facility (from November to May) was 9.9 (0.03) (min 1.7 max 22.4) and 6.6 (0.04) (min −5.2; max 22.6) for the ambient (outside) temperature.

#### Animal diet

The mean (±SE) chemical composition and nutritive value of the TMR offered to animals was: crude ash 81.4 g/kg DM (2.09), crude protein 149.8 g/kg DM (1.52), in vitro DM digestibility (DMD) 798.1 g/kg (4.03), neutral detergent fibre (NDF) 450.4 g/kg DM (5.0), pH 4.2 (0.03).

#### Animal cleanliness

There was a significant treatment, day and treatment × day interaction (P < 0.05) for dirt score (Table 2). Dirt scores did not differ (P > 0.05) between mat or slat treatments but were lower (P < 0.05) than for the OWP treatment with an overall significant treatment × day interaction (P < 0.001). Dirt scores were greater (P < 0.05) on OWPs on days 42, 84, 105, 126 and 150 compared with slats.

**Table 2:** Cleanliness scores of finishing beef steers on concrete slats, Mat 1, Mat 2, Mat 3 and OWP’s from day 0 to day 150. Values are expressed as Lsmeans (±SE)

	d 0	d 21	d 42	d 63	d 84	d 105	d 126	d 150	SE
Concrete slats	37^a^	45	48^x^	43	39^x^	34^x^	27^b,x^	23^b,x^	0.112
Mat 1	37^a^	45^b^	48	46	41	33	24^b^	21^b^	0.112
Mat 2	36^a^	47^b^	50^b^	46^b^	43	35	27^b^	22^b^	0.112
Mat 3	37^a^	48	51^b^	49^b^	44	34	26^b^	22^b^	0.112
OWP’s	35^a^	43	55^b,y^	42	57^b,y^	48^b,y^	40^y^	32^y^	0.112

Sum of 16 body parts each on a cleanliness scale of 1 (clean) to 5 (dirty)

Interactions; Treatment P = 0.006; Day P < 0.001; Treatment × day P < 0.001

^a,b^Lsmeans (in rows) that do not have a common superscript differ by P < 0.05

^x,y^Lsmeans (in columns) that do not have a common superscript differ by P < 0.05

#### Animal health, performance, and hoof lesions

No animal presented with clinical signs of bovine respiratory disease or required veterinary treatment for any ailment throughout the study period. The daily live weight gain of animals on the OWPs was greater for animals on the OWP compared with animals housed on slats and on Mat 2; Mat 1 and Mat 3 were not different from the other treatments (Table 3). However, carcass weight, kill out proportion, carcass fat score, carcass composition score and FCR were similar among treatments (Table 3). Animals on the OWPs had a greater (P < 0.05) dry matter (DM) feed intake compared with animals on the slat and the three mat types (Table 3). DMI was similar (P > 0.05) between the slat and mat treatments. The number of hoof lesions was greater on all mat types (P < 0.05) compared with concrete slats and OWP treatments (Table 3).

**Table 3:** Intake, performance, slaughter traits, hoof scores, behaviour of finishing beef steers housed on concrete slats, Mat 1, Mat 2, Mat 3 and OWP’s

	Concrete slats	Mat 1	Mat 2	Mat 3	OWP’s	SE	Significance
Initial weight (kg)	538.4	538.4	538.4	538.4	539.1	9.40	NS
Final weight (kg)	677.2	693.4	676.1	682.6	699.5	7.37	NS
Carcass (kg)	371.9	385.8	374.7	381.1	384.7	5.00	NS
Live weight gain/day	0.93^a^	1.04	0.92^a^	0.97	1.08^b^	0.03	P = 0.011
Kill out proportion (KO) (%)	55.0	55.7	55.4	55.8	55.1	0.40	NS
Kidney and channel fat (kg)	11.2	10.7	11.4	10.9	11.2	0.37	NS
Carcass composition score^1^	9.0	9.5	8.8	9.1	9.4	0.23	NS
Carcass fat score^2^	10.7	10.7	10.9	10.8	10.7	0.22	NS
Dry matter intake (DMI) kg/animal/day	9.1^a^	9.3^a^	9.2^a^	9.2^a^	10.1^b^	0.09	P < 0.001
DMI/LWG	9.9	9.0	10.0	9.5	9.5	0.34	NS
FCR (kg DMI/kg of LWG gained)^3^	9.9	9.0	10.0	9.5	9.5	0.35	NS
Hoof lesions^4^	20^a^	33^b^	32^b^	30^b^	24^a,b^	3.38	P = 0.050
Percent lying time (%)	49.4^a^	47.2^a^	48.7^a^	45.2^a^	38.0^b^	1.11	P < 0.001
Percent eating (%)	18.1^a^	20.6	18.0^a^	20.9	22.1^b^	0.94	P = 0.003
Percent drinking time (%)	0.60^a^	0.48	0.54^a^	0.51	0.38^b^	0.04	P = 0.010

Values are expressed as Lsmeans (SE)

*NS* not statistically significant

^a,b^Lsmeans (in rows) that do not have a common superscript differ by P < 0.05

^1^EU Beef Carcass Classification Scheme, scale 1 (poorest) to 15 (best)

^2^EU Beef Carcass Classification Scheme, scale 1 (leanest) to 15 (fattest)

^3^FCR was expressed as kilogram carcass gain per 1000 kg DMI

^4^The total number of hoof lesions/animal at the end of the study

#### Animal behaviour

Overall (period 1–6 combined), the percentage of animals lying at any one time, was greater (P < 0.05) in the animals housed on concrete slats, mats 1, 2 and 3 compared with the animals housed on the OWPs (Table 3). The percentage of animals eating at any one time was lower (P < 0.05) on concrete slats and Mat 2 compared with OWPs. The percentage of animals drinking at any one time was lower on OWPs compared with concrete slats and Mat 2 and was not different from Mat 1 and Mat 3.

#### Physiological variables

There was no effect of treatment or treatment × day interaction (P > 0.05) for plasma concentrations of albumin, total protein, βHB, NEFA, glucose, fibrinogen, haptoglobin, Con-A, PHA-induced IFN-γ and CK activity (Table 4). Day of sampling was significant (P < 0.05) for plasma concentrations of albumin, βHB, NEFA, glucose, fibrinogen, haptoglobin, PHA-induced-IFN-γ production and CK activity.

**Table 4:** Plasma metabolic variables (albumin, globulin, total protein, βHB, NEFA, Glucose, creatine kinase), acute phase proteins (fibrinogen, haptoglobin) and immune variables Concanavalin-A (Con-A) induced and Phytohaemagglutinin (PHA) induced interferon-γ (IFN-γ) of finishing beef steers on day 0 and day 150

	Bleed	Concrete slats	Mat 1	Mat 2	Mat 3	OWP	SE	Treatment (T)	Day (D)	T × D
Albumin	d 0	32.64^a^	32.44^a^	32.90^a^	32.56^a^	32.81^a^	0.143	NS	P < 0.05	NS
(g/l)	d 150	34.16^b^	34.63^b^	34.23^b^	34.54^b^	34.01^b^	0.192			
Globulin	d 0	41.43	41.58	41.83	41.68	42.19	0.515	NS	NS	NS
(g/l)	d 150	41.02	39.92	40.59	40.39	38.88	0.505			
Total protein	d 0	74.06	74.02	74.72	74.24	74.99	0.552	NS	NS	NS
(g/l)	d 150	75.18	74.55	74.82	74.93	72.88	0.456			
βHB	d 0	0.27^a^	0.27^a^	0.29^a^	0.27^a^	0.29	0.01	NS	P < 0.05	NS
(mmol/l)	d 150	0.23^b^	0.23^b^	0.23^b^	0.22^b^	0.28	0.01			
NEFA	d 0	0.15	0.17	0.17	0.19	0.22^a^	0.01	NS	P < 0.05	NS
(mmol/l)	d 150	0.16	0.18	0.15	0.16	0.13^b^	0.01			
Glucose	d 0	3.98^a^	3.93^a^	4.05^a^	4.01^a^	3.99	0.03	NS	P < 0.05	NS
(mmol/l)	d 150	4.29^b^	4.21^b^	4.30^b^	4.34^b^	4.12	0.03			
Creatine kinase	d 0	273.5^a^	255.8^a^	266.0^a^	244.0^a^	321.8^a^	11.86	NS	P < 0.05	NS
(U/l)	d 150	165.9^b^	183.3^b^	159.3^b^	166.3^b^	173.8^b^	7.18			
Fibrinogen	d 0	489.0^a^	483.2^a^	502.3^a^	498.6^a^	511.0^a^	11.04	NS	P < 0.05	NS
(mg/dl)	d 150	382.3^b^	366.0^b^	364.2^b^	348.1^b^	351.2^b^	8.46			
Haptoglobin	d 0	0.75^a^	0.75^a^	0.76^a^	0.77^a^	0.78^a^	0.02	NS	P < 0.05	NS
(g of Hb binding capacity/l)	d 150	0.64^b^	0.58^b^	0.62^b^	0.58^b^	0.55^b^	0.01			
Concanavalin-A (CON-A) induced IFN-γ	d 0	1.04	1.03	0.83	0.98	0.93	0.10	NS	NS	NS
Absorbance @450 nm	d 150	0.95	1.05	0.84	1.07	0.93	0.11			
Phytohaemagglutinin (PHA) induced IFN-γ	d 0	0.90^a^	0.83^a^	1.06^a^	0.91^a^	0.81^a^	0.09	NS	P < 0.05	NS
Absorbance @450 nm	d 150	0.48^b^	0.52^b^	0.59^b^	0.59^b^	0.58^b^	0.06			

Values are expressed as Lsmeans (SE)

*NS* not statistically significant, *T* Treatment, *D* Day, *T* *×* *D* Treatment × day interaction

^a,b^Lsmeans (in rows) that do not have a common superscript differ by P < 0.05

#### Haematological variables

There was no effect of treatment or treatment × day interactions (P > 0.05) whereas day (P < 0.05) was significant for basophil, eosinophil, leukocyte, lymphocyte, monocyte, neutrophil, red blood cell number, haemoglobin concentration and haematocrit percentage (Table 5). There was no effect of treatment, sampling day or treatment × day interaction (P > 0.05) for white blood cell number.

**Table 5:** Haematological variables of finishing beef steers on day (0) and d 150

	Bleed	Concrete slats	Mat 1	Mat 2	Mat 3	OWP	SE	Treatment (T)	Day (D)	T × D
WBCP	d 0	9.11	9.46	9.19^a^	9.61^a^	9.60	0.163	NS	P < 0.05	NS
(1 × 10^3^ µl)	d 150	8.85	9.03	8.66^b^	8.82^b^	9.82	0.166			
Basophil number	d 0	0.07^a^	0.08^a^	0.07^a^	0.08^a^	0.08^a^	0.002^a^	NS	P < 0.05	NS
(1 × 10^3^ µl)	d 150	0.06^b^	0.06^b^	0.06^b^	0.06^b^	0.06^b^	0.002^b^			
Eosinophil number	d 0	0.07^a^	0.08^a^	0.07^a^	0.08^a^	0.08^a^	0.002	NS	P < 0.05	NS
(1 × 10^3^ µl)	d 150	0.06^b^	0.06^b^	0.06^b^	0.06^b^	0.06^b^	0.002			
Leukocyte number	d 0	0.07	0.05	0.06	0.05	0.09^a^	0.005	NS	P < 0.05	NS
(1 × 10^3^ µl)	d 150	0.05	0.05	0.06	0.05	0.05^b^	0.003			
Lymphocyte number	d 0	5.29	5.24	5.11	5.58	5.47	0.098	NS	P < 0.05	P < 0.064
(1 × 10^3^ µl)	d 150	5.67	5.73^b^	5.34	5.76	6.00^b^	0.100			
Monocyte number	d 0	0.55^a^	0.57^a^	0.49^a^	0.54^a^	0.52^a^	0.014	NS	P < 0.05	P = 0.036
(1 × 10^3^ µl)	d 150	0.34^b^	0.38^b^	0.39^b^	0.37^b^	0.41^b^	0.009			
Neutrophil number	d 0	2.42	2.47^a^	2.37	2.68	2.33	0.054	NS	P < 0.05	NS
(1 × 10^3^ µl)	d 150	2.22	2.12^b^	2.29	2.35	2.14	0.046			
Haematocrit	d 0	33.39^a^	33.10^a^	33.22^a^	32.27^a^	33.72^a^	0.36	NS	P < 0.05	NS
%	d 150	37.60^b^	37.45^b^	36.62^b^	37.00^b^	36.44^b^	0.24			
Platelet number	d 0	447.9^a^	464.1^a^	484.2^a^	485.8^a^	457.3^a^	8.65^a^	NS	P < 0.05	NS
(1 × 10^3^ µl)	d 150	203.4^b^	185.2^b^	192.2^b^	205.9^b^	195.4^b^	30.39^b^			
Red blood cell number	d 0	8.19^a^	8.14^a^	7.98	7.87^a^	8.29	0.101	NS	P < 0.05	NS
(1 × 10^3^ µl)	d 150	8.62^b^	8.62^b^	8.32	8.46^b^	8.50	0.068			
Haemoglobin	d 0	12.95	12.84	12.86	12.61	13.21	0.149	NS	NS	NS
(g/dl)	d 150	13.36	13.27	12.98	13.15	13.08	0.139			

Values are expressed as Lsmeans (SE)

*NS* not statistically significant, *T* Treatment, *D* Day, *T* *×* *D* Treatment × day interaction

^a,b^Lsmeans (in rows) that do not have a common superscript differ by P < 0.05

## Discussion

In the present study, measuring the responses of animals to the different floor types, in terms of performance, hoof score, dirt scores, behaviour, immunological and physiological variables provided an insight into how the animals were coping. The objective of this study was to explore, in systematic fashion, factors affecting the overall performance, and welfare of animals housed on different floor types. The rationale for using TMR feeding was to achieve a relatively stable rumen pH and fermentation pattern throughout the day. This has been reported to promote better cellulose digestion and a greater lipogenic to non-lipogenic volatile fatty acid (VFA) ratio [31, 32]. Animals on the OWPs had a greater live weight gain compared with the slat and Mat 2 treatments: Mat 1 and Mat 3 were the same as the other treatments. Interestingly, carcass weight, kill out proportion, carcass fat score, carcass composition score and FCR were similar among treatments. The similar carcass weight for animals on the slat treatment in the present study is consistent with other studies in Ireland examining the performance and carcass characteristics of finishing animals housed on concrete slats and fed similar diets [32–38].

Dry matter intake was greater in the OWPs compared with all other treatments, whereas feed conversion ratio (FCR) was similar across treatments. Studies comparing concrete slats with straw as underfoot conditions in pens for finishing bulls are often confounded by space allowance, as housing systems utilising straw, have a larger total floor area per animal than fully slatted pens [39]. Gygax et al. [40] examined the effects of four space allowances (2.5, 3.0, 3.5 and 4.0 m^2^) on the behaviour and cleanliness of finishing bulls on fully slatted rubber coated floors and concluded that that increasing space allowance had several beneficial and no negative effects on welfare indicators. In the present study, dirt scores were greater on the OWPs compared with all other treatments. Additionally, animals on the OWPs spent less time lying compared with all treatments. It is reported in the literature that cattle housed on concrete floors tended to lie down or stand up less frequently and displayed more abnormal movements than animals housed on straw [2–4, 13, 18, 41, 42]. Bulls housed on slatted floors were reported to be more hesitant about lying down and were more likely to lie down via the “dog-sitting” position [43]. In addition, Graf [2] recorded a greater incidence of abnormal lying down movements among bulls on slatted concrete floors, but only at low space allowances.

A series of experiments examining the flooring preferences of cattle were conducted by Irps [44, 45]. In one study [44] 15-mo-old cattle spent more time lying on a straw-covered area than on rubber-coated concrete slats and spent the least time lying on uncovered concrete slats when they had access to all three areas. However, when lying times for fattening bulls were measured across three pens, one with rubber-coated concrete slats, one with 50% rubber-coated and 50% plain concrete slats and one with 100% plain concrete slats, there were no differences in lying times across the three systems [46]. Rubber coated slatted floors have been tested as an alternative floor quality in housing systems for finishing bulls [5, 39, 40] and the results indicate that finishing bulls lie down more frequently, slip less and have fewer hock lesions compared with fully slatted concrete floors. Interestingly, Elmore et al. [1] reported that flooring substrate had a large impact on health, hygiene, and postural changes of finishing beef steers, though there was no effect of flooring treatment on time budget behaviour or performance data. In the present study, the number of hoof lesions was greater on all mat types compared with concrete slats and OWP treatments. Although significant differences in numbers of lesions were found, the lesions were not severe enough to be biologically meaningful in terms of lameness. No information is available on the effects of high friction floors on cattle locomotion. Phillips and Morris [47–49] and Phillips et al. [50] measured these variables in cows walking on floors with different levels of friction. Phillips and Morris [49] and Phillips et al. [50] reported that floor friction had a considerable impact on the walking pattern of cows. Cows walked quickly with frequent, short steps on low friction floors (μ < 0.4). As μ increased to 0.5 (mean of static and sliding friction) with aggregates of 0.5 and 1.2 mm, step length increased and the number of steps decreased in order to maintain speed. Increased μ also may increase the hanging limb phase at the expense of the supporting limb phase, to reduce friction, while maintaining a long stride. Their findings suggest that the optimum coefficient of friction for cattle floors is between 0.4 and 0.5 and significantly Web and Nilsson [51] reported that the incidence of slip increases rapidly as coefficients of static friction for floors decrease below 0.4.

In the present study, the hardness values (Shore A scale measurements to ISO 7619) ranked the mats from hardest to softest (Mat 2, Mat 1, and Mat 3). The coefficients of friction were determined for each material in both wet and dry conditions on a roughened metal test track (an adaptation of ISO 15113). For both wet and dry tests, the ranking was Mat 2, Mat 1, and Mat 3 where the friction coefficients went from highest to lowest. In creep tests under constant compressive loading which replicated a typical stress resulting from the load transmitted under one hoof, the ranking order after load application was 3, 2, 1 (largest to smallest change in deformation). Mat 2 produced the highest coefficient of friction under both wet and dry conditions when subject to sliding motion in contact with a rigid surface. This is considered to be a positive characteristic in respect of comfort and safety and more likely to promote behaviour similar to that in the cow’s outdoor environment. If a high coefficient of friction, high static and dynamic deformation and low hardness were seen as desirable attributes for the mat materials and equal weighting is given to each property, there is little to choose between the performance of each mat.

Without devising specific tests, it is problematic to study the influence of dynamic and static friction when indentation of rubber takes place, as in the situation where a load is transmitted by a hoof. It is essential to minimise the influence of the dynamic properties of apparati if friction data is to be believed. Tests also need to quantify the contributions of bulk friction and adhesive friction [52]. Further research could shed light on these factors.

Alterations in the levels of blood cell constituents are indicative of an attempt to restore homeostasis when adverse physical conditions are encountered by an animal and thus, blood cells are very sensitive indicators of the patho-physiological responses of animals to a stressor [53].

In the present study, lymphocyte functional assays in terms of PHA-induced and Con A-induced IFN-γ production were used to assess cell-mediated immune function. The results showed no change in immune response among treatments in response to stimulation in vitro with the two mitogens, CON-A and PHA, indicating that there was no impairment of immune responsiveness of animals on the slat compared with the other treatments. Induction of a proliferative response induced by antigen in vitro has been shown to be representative of cellular immunocompetence [24]. The secretion of IFN-γ by lymphocytes is critically important in orchestrating an effective immune response, especially cellular immunity [29], and the induction of IFN-γ by Con A and PHA lectins corresponds to the degree of blastogenesis in cattle [54]. In the present study, maintenance of homeostasis was not affected by floor type. There was no effect of floor type on blood metabolite concentrations; thus metabolic homeostasis was maintained across treatments and the metabolite concentrations in the present study were within the normal physiological ranges for cattle [55–57].

## Conclusions

Steers on the OWPs had reduced lying percentage time compared with all the other treatments. Carcass weight, kill out proportion, carcass fat score, carcass composition score, and FCR, and physiological responses were similar among treatments. No incidence of laminitis was observed among treatments. There were a greater number of lesions on the hooves of animals housed on mats compared with bare concrete slats and OWP treatments. Therefore, under appropriate environmental conditions, out-wintering steers on OWP’s is a good alternative to wintering indoors on bare concrete slats. However, dirt scores were greater for animals on OWPs on days 42, 84, 105, 126 and 150 compared with slats. Under the conditions of the present study, there is no evidence to suggest that animals housed on bare concrete slats are at an animal welfare disadvantage compared with other floor treatments.

## Abbreviations

ADF: acid detergent fibre
bHB: beta hydroxy butyrate
CCTV: close circuit television. CK: creatine kinase
Con-A: concanavalin-A
DM: dry matter
DMD: dry matter digestibility
DMI: dry matter intake
IFN-γ: interferon-γ
Lsmeans: least square means
PHA: phytohaemagglutinin A
FCR: feed conversion ratio
NEFA: non-esterified fatty acids
NS: not statistically significant
RBC: red blood cell
SD: standard deviation
SE: standard error
TMR: total mixed ration
WBC: white blood cell
